# Investigating the Efficacy of an Individualized Alpha/Delta Neurofeedback Protocol in the Treatment of Chronic Tinnitus

**DOI:** 10.1155/2019/3540898

**Published:** 2019-03-26

**Authors:** Dominik Güntensperger, Christian Thüring, Tobias Kleinjung, Patrick Neff, Martin Meyer

**Affiliations:** ^1^Division of Neuropsychology, Department of Psychology, University of Zurich, Zurich, Switzerland; ^2^University Research Priority Program “Dynamics of Healthy Aging”, University of Zurich, Zurich, Switzerland; ^3^Department of Otorhinolaryngology, University Hospital Zurich, Zurich, Switzerland; ^4^Center for Neuromodulation, University of Regensburg, Regensburg, Germany; ^5^Tinnitus-Zentrum, Charité-Universitätsmedizin, Berlin, Germany

## Abstract

First attempts have demonstrated that the application of alpha/delta neurofeedback in the treatment of chronic tinnitus leads to a reduction of symptoms at the group level. However, recent research also suggests that chronic tinnitus is a decidedly heterogeneous phenomenon, one that requires treatment of distinct subgroups or even on an individual level. Thus, the purpose of this study was to evaluate an individually adjusted alpha/delta neurofeedback protocol. Following previous studies, the delta band fixed between 3 and 4 Hz was chosen as the frequency for inhibition. However, unlike the previous studies, the frequency range for the rewarded alpha band was not fixed between 8 and 12 Hz but rather individually determined according to each patient's specific alpha peak frequency (IAF). Twenty-six chronic tinnitus patients participated in 15 weekly neurofeedback training sessions and extensive pre- and post-tests, as well as follow-up testing 3 and 6 months after training. The main outcome measures were tinnitus-related distress measured with the Tinnitus Handicap Inventory (THI) and Tinnitus Questionnaire (TQ), tinnitus loudness, and pre- and post-training resting-state EEG activity in trained frequency bands. In Results, the neurofeedback protocol led to a significant reduction of tinnitus-related distress and tinnitus loudness. While distress remained on a low level even 6 months after the completion of training, loudness returned to baseline levels in the follow-up period. In addition, resting-state EEG activity showed an increase in the trained alpha/delta ratio over the course of the training. This ratio increase was related to training-induced changes of tinnitus-related distress as measured with TQ, mainly due to increases in the alpha frequency range. In sum, this study confirms the alpha/delta neurofeedback as a suitable option for the treatment of chronic tinnitus and represents a first step towards the development of individual neurofeedback protocols. This clinical trial was registered online at ClinicalTrials.gov (NCT02383147) and kofam.ch (SNCTP000001313).

## 1. Introduction

Approximately 5-15% of the Western population suffers from a permanent sensation of ringing or hissing in their ears, a phenomenon also known as chronic subjective tinnitus [1]. According to Henry et al. [1], around 20% of affected people suffer considerably from this constant perception of sound which, in some cases, can result in a substantial reduction of quality of life. Often, chronic tinnitus can induce related issues, some of which include problems sleeping or concentrating, experiencing difficulty in social interactions, and perhaps even resulting in severe depression or anxiety [2–4]. An effective treatment to completely alleviate the symptoms of tinnitus has not yet been discovered, and thus, many sufferers do not receive the help that they need. As a consequence, this lack of sustained and effective intervention can lead to increased levels of stress and frustration which, in turn, compound the negative impact of tinnitus on the quality of life for many patients [5].

While in early research subjective tinnitus was assumed to be a problem associated with the peripheral hearing system only [6, 7], the currently widely accepted view is that this auditory phantom percept emerges as a result of unsuccessful compensatory mechanisms in the brain in consequence of inner ear receptor damage [8–11]. Further to this, electrophysiological recordings with electroencephalography (EEG) and/or magnetoencephalography (MEG) have led to the recognition of tinnitus-related abnormalities in spontaneous resting-state brain activity. According to recent studies in which the resting-state activity of tinnitus patients and healthy controls was compared, the resting brain of tinnitus patients typically shows enhanced activity in the delta (0.5-4 Hz) and gamma (35.5-45 Hz) frequency bands and a comparative reduction of alpha (8.5-12 Hz) oscillations over temporal areas [11–18]. The theoretical frameworks on which these findings are based are the thalamocortical dysrhythmia (TCD) model [19] and the synchronization-by-loss-of-inhibition model (SLIM) [11]. The TCD model describes the emergence of spontaneous firing of thalamic fibers due to auditory input deprivation as an essential factor for tinnitus genesis [19]. Specifically, when thalamic relay cells are deprived of excitatory sensory input from the inner ear, the hyperpolarized cell membrane causes these neurons to fire low-threshold calcium spike bursts in a slow-wave mode. Thalamocortical feedback loops then lead to the establishment of this slow-wave rhythm in cortical neurons, which is measurable as ongoing delta activity on the scalp. Llinás et al. [19] further propose that an *edge effect* resulting from these increased gamma oscillations is responsible for perceptive disturbances, such as tinnitus. Furthermore, it is suggested in the SLIM that this increase in the gamma frequency range may also be driven by decreased lateral inhibition processes in auditory cortex areas due to an underactivation of inhibitory neurons [11]. This imbalance between cortical inhibition and excitation thus provides a theoretical explanation for the alpha-down, delta-up pattern typically found in the resting-state M/EEG data of tinnitus patients [20].

Recently, neurofeedback has received increasing attention regarding its potential in the treatment of a variety of psychological and neurological disorders. In the process of neurofeedback, electrophysiological brain activity is recorded noninvasively, immediately analyzed in real-time, and certain aspects of it (e.g., frequency band power) extracted, which are then directly used for feedback to the subject. The rewarding of desired changes and inhibiting of undesired changes in the signal pattern by providing directly perceivable visual, auditory, and/or tactile feedback is proposed to trigger a learning process during which the patients learn to voluntarily control their brain activity and to adjust it in the desired direction. Neurofeedback has been in development since the late 1960s [21, 22] and is currently an established treatment method for attention deficit hyperactivity disorder (ADHD) [23–27]. Furthermore, first attempts have already been made to implement it as an effective treatment for chronic tinnitus (for a review, see [28]). In this context, the training of frequency bands linked to the aforementioned abnormalities in resting-state brain activity has been shown to be a highly promising approach. Two research groups reported that neurofeedback training aimed at increasing alpha and decreasing delta activity over auditory areas led to significant reductions in tinnitus-related symptoms (i.e., tinnitus distress and loudness) and that these behavioral changes were also linked to the trained resting-state activity [29, 30]. The gamma frequency band, however, has been largely neglected in neurofeedback treatments for chronic tinnitus. The reason for this is based on current debate, namely, that activity in the gamma band may reflect an attempt of the brain to suppress tinnitus rather than cause it [31, 32] or may be involved in the communication of prediction errors [33]. Given these inconsistencies, the inclusion of gamma oscillations in neurofeedback protocols for the treatment of tinnitus is unsuitable until their specific role is better understood. Furthermore, the usability of gamma for neurofeedback protocols is limited by its rather broad and unspecific bandwidth and a decreasing signal-to-noise ratio for higher frequencies.

The aim of this clinical study was thus to contribute to the development of effective neurofeedback protocols for tinnitus patients and to build on as well as extend the previously applied auditory alpha/delta training. For the recording of brain activity used for the feedback, the same EEG electrodes (FC1, FC2, F3, and F4) were chosen as in the previously mentioned studies [29, 30] to guarantee comparability. Regarding the frequency bands used for the training, however, we chose a novel approach. This was based on the recognition that chronic tinnitus is a very multifaceted and complex phenomenon, as noted in recent studies (e.g., [10, 34]). For this reason, we considered it extremely important to conceive the applied neurofeedback treatment on an individualized basis, thereby attempting to meet the specific needs of each tinnitus patient. This project takes the first step in this direction. In particular, we took into account observations that the individual alpha peak frequency (IAF) can vary considerably among individuals [35]. Using the fixed alpha band (generally defined between 8 and 12 Hz) for power analysis, therefore, does not reflect alpha band power for each subject appropriately. We believe that these interindividual differences should be considered when alpha is targeted in a neurofeedback training protocol. Further to this argument, a recent study with tinnitus patients has underlined the importance of taking the interindividual alpha variability into account for this group [36]. Based on this reasoning, we did not choose the standard alpha band (8-12 Hz) as a fixed reward frequency for each patient, which has customarily been the case in previous studies. Instead, an individual alpha peak frequency was determined for each tinnitus patient before the first neurofeedback session and an individually adjusted alpha band was then used for the generation of the neurofeedback reward.

In addition, we placed great emphasis on efforts to make our results replicable and comparable to other studies. Accordingly, we designed our study closely following the guidelines of the Tinnitus Research Initiative (TRI) on outcome measures for tinnitus intervention studies [37, 38]. We combined our training with a wide variety of questionnaires and tests at different time points while also using different measurements for tinnitus-related distress and other health-related variables. In addition, the classical pre-post design, generally used in treatment studies, was supplemented by two follow-up measurements in order to investigate longevity and persistence of the potential effects. The main behavioral outcome measures of this study were tinnitus-related distress, measured with two well-established tinnitus questionnaires, and tinnitus loudness. Both variables were hypothesized to decrease over the course of the neurofeedback training and to remain on a stable lower level at the follow-up time points. Furthermore, in order to examine whether the neurofeedback training indeed evoked the desired effects in EEG activity, the ratio between the rewarded alpha- and the inhibited delta band was compared across time points. It was expected that the alpha/delta ratio would change significantly between pre- and post-tests and would remain on a stable level in the follow-up period.

## 2. Methods

### 2.1. Participants

Participants were recruited at the Department of Otorhinolaryngology (University Hospital Zurich). In order to be eligible for study inclusion, patients had to be diagnosed with chronic subjective tinnitus (>0.5 years), be between 18 and 75 years old, have adequate knowledge of the German language, suffer from no other psychiatric or neurological disorder, and have no acute suicidal tendency. Furthermore, patients with drug or alcohol addiction, cochlear implants, and current prescriptions for tranquilizers, neuroleptics, or antiepileptics were not considered. It should be mentioned that this study is part of a comprehensive clinical project, and participants were randomly assigned to one of two study groups (single-blind randomized controlled trial). Both groups underwent the exact same procedure (see Section 2.2) with the sole difference being a technical aspect of feedback generation. The group reported here followed the neurofeedback application closely related to prior studies (see Section 2.5) in which the activity included for calculating reward and inhibit rates was limited to four electrodes. The other group used a marginally different approach in that more EEG electrodes in addition to source estimation algorithms were involved in feedback generation. The results of this group as well as between-group comparisons will be discussed elsewhere. According to the aforementioned criteria, 26 suitable patients with chronic subjective tinnitus were identified and included. Participants were between 24 and 71 years old with a mean age of 46.15 (SD: 12.33). The sample consisted of 20 males and 6 females. The study was approved by the appropriate Ethics Committee (Kantonale Ethikkommission Project KEK-ZH-Nr. 2014-0594) and was registered online at ClinicalTrials.gov (NCT02383147) and kofam.ch (SNCTP000001313).

### 2.2. Procedure

This prospective clinical trial consisted of 20 visits in total. In the first appointment, 1-2 weeks before the start of the neurofeedback training phase, patients were extensively informed about the purpose and exact procedure of the study and signed their informed consent in the presence of a qualified medical professional at the Department of Otorhinolaryngology. In the same visit, participants further underwent the audiometric screening in which their pure tone hearing thresholds at 0.25, 0.5, 1, 2, 4, 6, and 8 kHz as well as other audiometric measurements (speech audiogram and speech-in-noise test) were determined. In the second screening visit, a baseline resting-state EEG measurement was performed and patients were asked to complete questionnaires covering demographics and tinnitus-related symptoms, as well as several other psychological and health-related questions (details in Section 2.3).

After the two baseline appointments (t1), patients participated in a total of 15 neurofeedback training sessions on a weekly basis. Occasional rescheduling of individual sessions as well as absences due to holidays or illness was unavoidable and compensated for as best as possible. One week after the completion of the training period, a post-measurement was performed (t2) consisting of the repeated measurement of 16 minutes of resting-state EEG and completion of the questionnaires. The same procedure was repeated approximately 3 months later after the first follow-up measurement was conducted (t3). In the final follow-up (t4), 6 months after the end of the training period, patients received a link by email and were asked for another completion of the set of questionnaires online. Subsequently, they were informed that they had fully completed the clinical study and were provided the opportunity to discuss their individual results with the study team.

### 2.3. Behavioral Measurements

The set of questionnaires consisted of a variety of forms according to the guidelines of the Tinnitus Research Initiative (TRI) [37, 38]. Specifically, an adjusted version of the Tinnitus Sample Case History Questionnaire (TSCHQ) was used to ask about demographics, tinnitus properties (e.g., origin, location, loudness, and type), prior treatment attempts, and other tinnitus-related issues. Two questionnaires were used to assess tinnitus distress: the Tinnitus Handicap Inventory (THI) (German version by [39]) and the Tinnitus Questionnaire (TQ) (German version by [40]). Sum scores can be calculated for both questionnaires ranging from 0 to 100 in the former and 0 to 84 in the latter case. In addition, the TQ score can be divided into the six subscores: “emotional distress,” “cognitive distress,” “intrusiveness,” “auditory perceptual difficulties,” “sleep disturbances,” and “somatic complaints.”

Additionally, participants completed German versions of Beck's Depression Inventory (BDI) [41], Beck's Anxiety Inventory (BAI) [42], the short form of the WHO Quality of Life scale (WHOQOL-BREF) [43], Symptom Check List (SCL-K-9) [44], and Short Form Health Questionnaire (SF-36) [45]. Completion of questionnaires took about 45 minutes in total and was done electronically on an iPad during the preparation of the EEG system at t1, t2, and t3 and online via an email link at t4.

The main behavioral outcome measures of this study are tinnitus loudness (rated from 1 “very low” to 100 “very high”), sum score of the THI, and sum- as well as subscores of the TQ.

### 2.4. EEG Recording

A BrainAmp DC amplifier system in combination with 64 active channel actiCap electrode caps (Brain Products, Munich, Germany) were used to record the resting-state EEG at t1, t2, and t3. The array of silver/silver chloride electrodes corresponded with the 5/10 electrode position system [46]. Recording was referenced against the FCz electrode with a ground electrode positioned at the AFz position. A sampling rate of 1000 Hz was used. The electrodes were prepared with conductive paste for recording, and impedance was kept below 10 kΩ. Recordings were done in direct current (DC) mode with a high-cutoff filter of 1000 Hz with a slope of 12 dB/octave. Patients were asked to sit upright on a comfortable chair in a sound-proof and electromagnetically shielded room and to avoid excessive movements and muscle contractions in order to minimize artifacts. During recording, subjects were instructed by a prerecorded voice to open (EO) and close (EC) their eyes in regular intervals. For playback of these instructions, Presentation software (Neurobehavioral Systems Inc., 2010) was used and a fixation cross was presented during eyes-open segments.

Resting-state EEG was recorded twice over a time span of 8 minutes. While in the first 8 minutes of recording no additional instructions were given (EEG with no task: EEG-NT), in the second measurement, patients were asked to deliberately not suppress their tinnitus (EEG with task: EEG-WT). This was done to control for unwanted suppression effects that happen continuously in the brains of tinnitus sufferers (see also [31]). According to the recommendations of Working Group 3 of the European tinnitus research network, TINNET (http://www.tinnet.tinnitusresearch.net/), the resting-state activity of eyes-open segments was chosen as the main electrophysiological outcome measure.

### 2.5. Neurofeedback Training

EEG for neurofeedback training was registered with four silver/silver chloride electrodes, FC1, FC2, F3, and F4 combined with a NeuroAmp amplifier (BEE Medic GmbH, Singen, Germany). Electrodes at the earlobes served as reference electrodes and AFz as the ground electrode. The sampling rate was set at 250 Hz and impedance kept below 20 kΩ. The EEG signal was processed in real-time using the software Cygnet 2.0.3.34 (EEG Info, Kirchberg, Switzerland), and the feedback was implemented in the computer simulation Inner Tube (Somatic Vision, Encinitas, CA, USA). In this visualization, patients observed a space ship automatically navigating through a narrow tunnel. While increased power in the alpha band led to acceleration of the ship, delta as the defined inhibited band was linked to autopilot accuracy. It is important to note that automatic filtering is included in the Cygnet software so that excessive movement artifacts (blinking included) as well as system voltage (45-55 Hz) are automatically detected and excluded from feedback.

In the first neurofeedback training session, an individual alpha peak was determined for each participant by averaging alpha peaks over 30 seconds of resting-state EEG [35]. Subsequently, the reward frequency was set in the range of *±*2 Hz around this peak frequency. As the undesired alternate, the frequency range of 3-4 Hz corresponding to the delta band was generally set to evoke negative feedback. Patients were asked to sit comfortably in a chair, avoid excessive muscle movement, and pay close attention to the feedback game. Following the custom of previous studies [29, 30], no further instruction was given as to how to influence the feedback or what strategy to use in order to allow for the highest amount of freedom possible. The training itself lasted 15 minutes and was repeated once a week, preferably on the same weekday at the same time.

### 2.6. Data Analysis

#### 2.6.1. EEG Preprocessing

Preprocessing of EEG data was done with the BrainVision Analyzer 2 (Brain Products, Munich, Germany). Data was first band-pass filtered with Butterworth zero-phase filters between 0.1 Hz and 80 Hz with slopes of 24 dB/octave at the low and 48 dB/octave at the high cutoffs. In order to eliminate possible line noise, data was further filtered with a band-rejection filter with a central frequency of 50 Hz, a bandwidth of 1 Hz, and a slope of 24 dB/octave. The EEG signal was split into independent components in order to identify regular artifacts (e.g., eye blinks, pulse artifacts, noise). This was done by applying an independent component analysis (ICA) with a restricted Infomax algorithm implemented in BrainVision Analyzer 2. Bad channels (i.e., very noisy or dead channels, as well as electrodes with channel jumps) were temporarily excluded from this step. With the inverse ICA procedure, the resulting components indicative of artifacts were removed from the data. Subsequently, spline-type topographical interpolations [47] were performed for previously excluded channels and channels with remaining noise. On average, 5.4 components have been excluded and 1.9 channels interpolated per data set. A limit of ten bad channels (~15%) was priorly defined to lead to data set exclusion which was not the case for any of the data sets. A thorough visual inspection was performed in order to remove the remaining vertical artifacts (i.e., muscle movements and short drifts or jumps over single or multiple electrodes) from the signal. An average reference over all channels was calculated and applied whereby the implicit reference of data recording (FCz) was reincluded into the data and used for subsequent analysis. Finally, data was segmented into eyes-closed and eyes-open conditions and imported to MATLAB Statistics Toolbox Release 2017a (The MathWorks Inc., Natick, Massachusetts, United States) and EEGLAB 14.1.1b [48].

#### 2.6.2. EEG Analysis

A hamming window with 2 s window length and 1 s overlap was first applied on the data of eyes-closed and eyes-open segments. Subsequently, Fast Fourier Transform (FFT) was computed for each 2 s segment, logarithmized, and then averaged over all segments for each patient. The resulting values provided power values in decibel (dB) for each electrode of each measurement (EEG-NT and EEG-WT). The frequency resolution was thus 0.5 Hz. Next, we calculated the alpha/delta ratio by dividing power values in the rewarded (individual) alpha range by those in the inhibited delta range (3-4 Hz). This ratio was finally averaged over the four electrodes used for training (FC1, FC2, F3, and F4) as well as over all 65 electrodes of the EEG system. In addition, power values in the standard frequency bands delta (0.5-4 Hz), theta (4.5-8 Hz), lower alpha (8.5-10 Hz), upper alpha (10.5-12 Hz), alpha (8.5-12 Hz), beta1 (12.5-15 Hz), beta2 (15.5-23 Hz), beta3 (23.5-35 Hz), and gamma (35.5-45 Hz) were calculated and analyzed.

#### 2.6.3. Statistics

Data was analyzed using the software package R [49] including packages “ggplot2” [50], “ggsignif” [51], “Hmisc” [52], “jtools” [53], “multcomp” [54], “nlme” [55], and “xtable” [56]. Repeated-measure mixed model analysis of variance (ANOVA) was used to estimate time effects for behavioral (THI sum score, TQ sum- and subscores, and tinnitus loudness) and EEG-related data. A priori defined contrasts comparing t1 with all other time points (t2, t3, and t4 for behavioral measures; t2 and t3 for EEG data) were calculated to gain insight into training success and the stability of changes in the follow-up period. Since contrasts are not independent, Bonferroni correction was applied, and because the contrasts were set a priori, one-tailed *p* values are reported. Furthermore, effect size *r* for a priori defined contrasts is reported which was converted from respective *t* values according to Field et al. [57]. Cohen [58] suggests that *r* = 0.1 may be labelled a small, *r* = 0.3 a medium, and *r* = 0.5 a large effect. In addition, post hoc Tukey tests were performed comparing each of the four time points with each other in order to reveal other potential differences between time points. In order to test for relationships between changes in the behavioral and electrophysiological domain, Pearson product-moment correlation coefficients between difference scores (t2-t1) were calculated and tested for statistical significance. The alpha threshold was set at *p* = .05 for all statistical tests.

## 3. Results

### 3.1. Description of Study Sample

Two patients who completed the full study procedure had to be excluded prior to data analysis because their BDI scores at all four time points suggested clinically relevant depressive symptoms (i.e., a sum score of more than 18 points). The final sample size for data analysis was therefore reduced to 24 participants.  Table 1 shows the demographic and clinical details of the participants included in the final analysis. The study sample had a mean age of 46.29 (SD = 12.22) and consisted of 19 males and 5 females. All participants were right-handed. The percept was described mostly as tonal (*n* = 17) with a pitch described as “very high” in 12 subjects. Almost all (*n* = 21) subjects perceived tinnitus in both ears; however, 9 subjects of this group indicated a left- while 6 specified a right-sided tendency. Stress was named as the primal cause of tinnitus by 6 participants, 4 indicated acoustic trauma or hearing loss to be responsible, while the majority (*n* = 13) could not name an unambiguous cause for the condition.

For the overall group on average, the mean distress value of 29.33 (SD = 14.7) suggested a “mild tinnitus” according to the THI, while the mean TQ value of 23.75 (SD = 11.63) is labelled a “slight tinnitus.” It is important to note that all tinnitus distress and loudness measures were significantly positively correlated (THI and TQ: *r*(22) = 0.8, *p* < .001; THI and loudness: *r*(22) = 0.47, *p* = .022; TQ and loudness: *r*(22) = 0.56, *p* = .004).

Pearson correlations between tinnitus- and health-related measures are summarized in Table 2. All correlations are corrected for multiple comparisons using the method of Benjamini and Hochberg [59]. Notably, depressive symptoms as measured with the BDI were positively correlated with THI, *r*(22) = 0.75, *p* < .001, as well as TQ sum scores, *r*(22) = 0.79, *p* < .001, but not loudness, *r*(22) = 0.48, *p* = .052. Furthermore, significant negative correlations were observed between quality of life as measured with the psychological health domain of the WHOQOL-BREF (domain 2) and all tinnitus measures (THI: *r*(22) = -0.63, *p* = .004; TQ: *r*(22) = -0.55, *p* = .021; loudness: *r*(22) = -0.52, *p* = .029). Moreover, significant negative correlations were found between the mental health score of SF-36 and THI, *r*(22) = -0.69, *p* = .002, and TQ sum scores, *r*(22) = -0.66, *p* = .003.

### 3.2. Effects of Neurofeedback Training

#### 3.2.1. Main Outcomes

**Figure 1:**
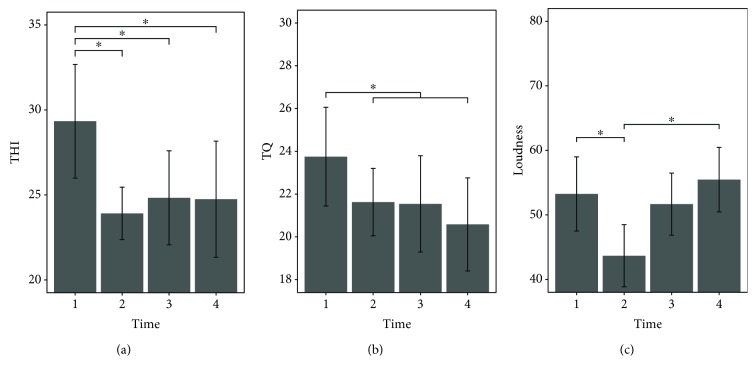
Barplots showing tinnitus-related symptoms before (t1), 1 week after (t2), 3 months after (t3), and 6 months after (t4) neurofeedback training. Error bars represent ±1 standard error for within-subject designs according to Morey [60]. THI scores (a) showed significant decreases from t1 to t2, and differences between t1 and the two follow-up time points were significant. TQ scores (b) were significantly higher before (t1) than after the neurofeedback intervention (t2-t4). For tinnitus loudness (c), a significant decrease between t1 and t2 was found followed by a significant increase to t4.

**Figure 2:**
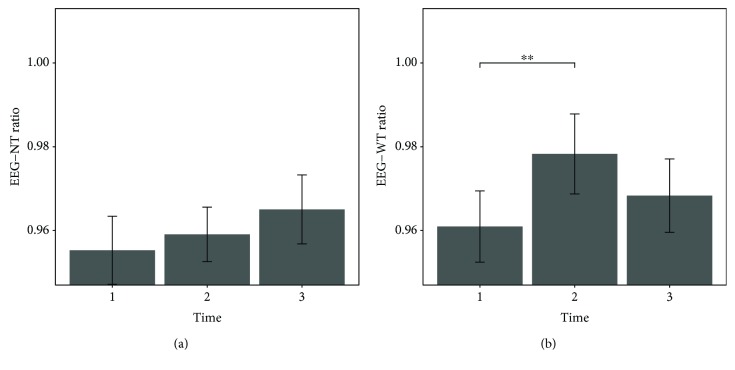
Barplots showing the alpha/delta power ratio over the four electrodes used for training in measurements before (t1), 1 week after (t2), and 3 months after (t3) the neurofeedback intervention. Error bars represent ±1 standard error for within-subject designs according to Morey [60]. The alpha/delta ratio of EEG-NT (a) did not vary significantly over time. The ratio of EEG-WT (b) increased significantly over the course of the training, between t1 and t2, followed by a nonsignificant decrease to t3.

Primary outcome variables across the four time points are presented in Table 3, as well as Figures 1 and 2. Results of the repeated-measure mixed model ANOVA as well as a priori defined contrasts are summarized in Table 4.

The repeated-measure mixed model ANOVA suggested significant effects of the factor *time* on tinnitus-related distress measured with the THI, *χ*^2^(3) = 9.18, *p* = .027, and tinnitus loudness, *χ*^2^(3) = 12.4, *p* = .006. Results for the TQ, on the other hand, did not suggest significant differences over time, *χ*^2^(3) = 5.24, *p* = .155. However, an ANOVA performed on the subscores of TQ revealed significant time effects for “emotional distress,” *χ*^2^(3) = 8.94, *p* = .03.

A priori defined contrasts for THI-measured distress showed significant decreases between t1 and the other 3 time points (see Table 4). A post hoc Tukey test corroborated these three significant results and revealed no further significant differences. It is important to note that, even though the main analysis for TQ did not reveal a significant effect, the sum score measured prior to the neurofeedback training at t1 (*M* = 23.75, SD = 11.63) was found to be significantly higher than the average over the three time points after neurofeedback (*M* = 21.25, SD = 12.01), *t*(69) = −2.14, *p* = .018 (one-tailed). In the case of TQ, no other significant differences were found with the Tukey post hoc test.

For rated tinnitus loudness, a priori-defined contrasts revealed a significant decline between t1 (*M* = 53.25, SD = 19.57) and t2 (*M* = 43.67, SD = 22.42), *t*(69) = −2.74, *p* = .012 (one-tailed). However, the Tukey test further revealed a significant increase between t2 and t4 (*M* = 55.46, SD = 17.28), *p* = .003, suggesting a recession of the rated tinnitus loudness to the baseline value, 6 months after the training.

Regarding EEG data, the repeated-measure mixed model ANOVA suggested a significant effect of the factor *time* for the EEG with the instruction to focus on the tinnitus percept (EEG-WT), *χ*^2^ (2) = 7.77, *p* = .021. The alpha/delta ratio of the resting-state measurement without instruction (EEG-NT) did not vary significantly over time, *χ*^2^(2) = 3.54, *p* = .17. For EEG-WT, the alpha/delta ratio showed a significant increase between t1 (*M* = 0.961, SD = 0.0422) and t2 (*M* = 0.9783, SD = 0.0443), *t*(46) = 2.83, *p* = .007 (one-tailed). This increase was followed by a slight decrease measured 3 months after the training, which was non-significant as Tukey tests, besides t1-t2, did not show any meaningful differences between time points. The contrast analysis for EEG-NT did not reveal any significant results.

When the individual alpha band as the reward frequency and the 3-4 Hz fixed delta band as the inhibit frequency of the neurofeedback training were compared separately across time, none of the repeated-measure ANOVAs suggested a significant time effect (see Table 4). Nonetheless, contrast analysis revealed a significant decrease in the trained delta band of EEG-WT over the course of the training between t1 (*M* = 51.87, SD = 1.86) and t2 (*M* = 51.18, SD = 1.92), *t*(46) = −2.42, *p* = .02 (one-tailed).

#### 3.2.2. Control Comparisons

To control for band specificity of the neurofeedback training, separate analyses were performed for the other (non-trained) frequency bands: theta, beta1, beta2, beta3, and gamma. In addition, the standard bands delta and alpha were analyzed according to their traditional definitions of frequency boarders (see Section 2.6.2) instead of the ones used for neurofeedback in this study (3-4 Hz for delta and the individual range for alpha). The alpha band was further subdivided into a lower and an upper alpha band according to standard conventions.

Apart from the standard delta band, the ANOVAs for the two EEG conditions (EEG-NT and EEG-WT) did not suggest any significant effects of the factor *time* on these untrained frequency bands and none of the performed contrasts nor the Tukey post hoc tests showed significant differences between time points.

Secondly, topographical specificity of the neurofeedback protocol was investigated. In order to assess whether the effects described in the previous section were restricted to the four electrodes used in the training, time effects of the trained alpha/delta ratio averaged over all 65 electrodes of the EEG system were analyzed. Repeated-measure mixed model ANOVA suggested significant effects of the factor *time* for both EEG conditions (EEG-NT: *χ*^2^(2) = 9.67, *p* = .008; EEG-WT: *χ*^2^(2) = 9.6, *p* = .008). For the measurement without instruction (EEG-NT), contrasts only suggested a significant ratio increase between t1 (*M* = 0.9636, SD = 0.0433) and t3 (*M* = 0.9786, SD = 0.042), *t*(46) = 3.2, *p* = .002 (one-tailed). In the case of EEG-WT, both contrasts showed significant results and meaningful differences were found between t1 (*M* = 0.9703, SD = 0.0441) and t2 (*M* = 0.9861, SD = 0.0457), *t*(46) = 3.1, *p* = .003 (one-tailed), as well as between t1 and t3 (*M* = 0.9815, SD = 0.0443), *t*(46) = 2.2, *p* = .033 (one-tailed). Tukey post hoc tests confirmed these findings and suggested no further significant differences.

Finally, in order to determine whether the potential effects of the neurofeedback intervention are limited to a certain age group, a control analysis has been performed. The 24 tinnitus patients included in this study have been subdivided into two subgroups according to their age. This was done by means of a median split on the variable *age* (Mdn = 44). Accordingly, 13 patients have been assigned to a young and 11 cases to an old group. When including this control factor in the repeated-measure mixed model ANOVA as an interaction term, none of the models showed an increased fit on the data (see Table 5).

#### 3.2.3. Correlations

**Figure 3:**
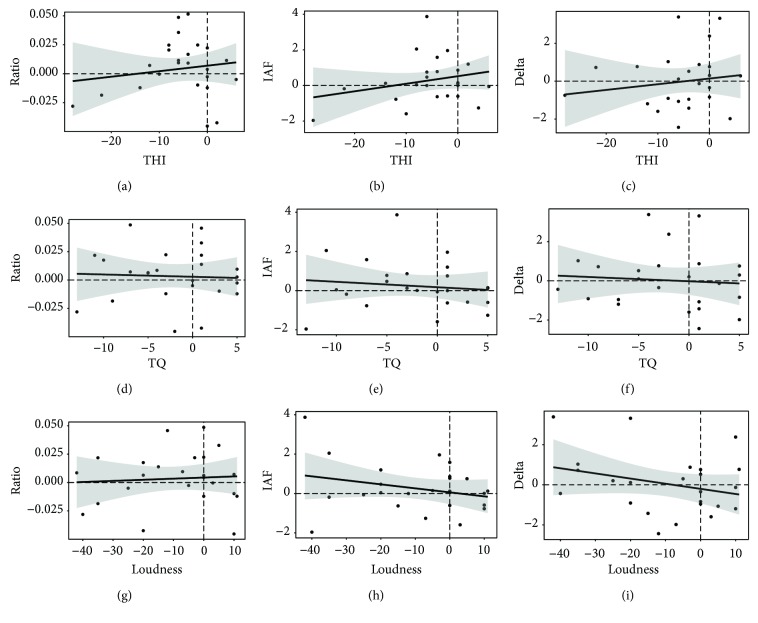
Scatterplots of difference scores (t2-t1) of EEG-NT resting-state data ((a, d, g) alpha/delta ratio; (b, e, h) rewarded individual alpha band power; (c, f, i) inhibited delta band power) and tinnitus-related symptoms ((a–c) THI; (d–f) TQ; (g–i) tinnitus loudness). The plots show the fitted regression lines with 95% confidence intervals. No correlations were found to be statistically significant.

**Figure 4:**
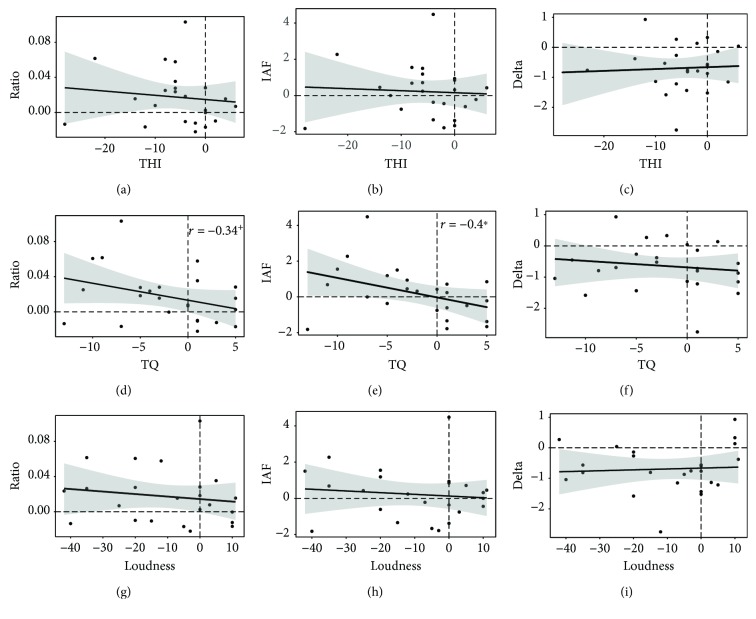
Scatterplots of difference scores (t2-t1) of EEG-WT resting-state data ((a, d, g) alpha/delta ratio; (b, e, h) rewarded individual alpha band power; (c, f, i) inhibited delta band power) and tinnitus-related symptoms ((a–c) THI; (d–f) TQ; (g–i) tinnitus loudness). The plots show the fitted regression lines with 95% confidence intervals. The correlation between IAF and TQ difference scores is statistically significant (*p* > .05).

To investigate the relationship between training-induced behavioral and electrophysiological changes, difference scores (t2-t1) in the two domains were calculated and compared. Pearson product-moment correlations are summarized in Table 6, as well as in Figures 3 and 4. Changes in the alpha/delta ratio correlated with THI differences with *r*(22) = 0.12 for EEG-NT and with *r*(22) = -0.12 for EEG-WT. None of these correlations reached statistical significance. Also, for TQ, the negative Pearson correlation coefficient for EEG-NT did not reach statistical significance, *r*(22) = -0.03, *p* = .449 (one-tailed). On the other hand, difference scores of the alpha/delta ratio of EEG-WT suggested a statistical trend for a negative correlation, *r*(22) = -0.34, *p* = .053 (one-tailed). Notably, when analyzed separately, a significant negative correlation was found between the changes in the trained individual alpha frequency band and TQ sum score differences, *r*(22) = -0.4, *p* = .026 (one-tailed). No significant relationships were found for the trained frequency bands and changes in tinnitus loudness.

## 4. Discussion

The neurofeedback protocol used in this clinical study aimed at alpha-up, delta-down training with an individualized alpha reward frequency range determined for each patient. It is fair to say that the chronic tinnitus patients who participated in this study benefited greatly from the neurofeedback intervention as tinnitus-related distress measured with two different questionnaires (THI and TQ) decreased over the course of training. Furthermore, this decrease in distress was stable and remained on a lower level at both the 3- and 6-month follow-up evaluations. Tinnitus loudness was also found to be significantly decreased due to neurofeedback application. However, unlike tinnitus distress, loudness of the phantom percept increased again after the training was completed and returned to baseline levels in the follow-up period. It is important to note that patients did not report any severe and persisting side effects due to the neurofeedback application.

In line with these results, the two previous neurofeedback studies that worked with comparable protocols also reported improvements for tinnitus-related distress, as TQ values [30] as well as THI sum scores [29] were significantly diminished after the training and remained stable 6 months after completion of the training period. We were able to replicate these findings in our study. However, in both preceding studies, a stable recession for tinnitus loudness was also reported, which was not the case in our investigation since loudness was decreased only temporarily. A possible explanation for this inconsistency might be the higher frequency and length of neurofeedback sessions in these previous reports. While participants in our study underwent 15 minutes of neurofeedback training on a weekly basis, Dohrmann et al. [30] and Crocetti et al. [29] worked with 30 and 20 minutes, respectively, 2-3 times per week. Frequency and length of the training sessions might thus be considered a crucial factor for longer-lasting effects regarding tinnitus loudness.

In what follows, we discuss the most relevant implications that emerge from the comparison of our study with the previous reports. Included in the discussion will be the careful examination of whether data obtained within the scope of this project can support the hypothesis that our neurofeedback application led to specific training effects or whether these can be explained as the result of an unspecific placebo effect.

### 4.1. Analysis of Electrophysiological Data

Electrophysiological data has been analyzed in order to reveal whether the neurofeedback protocol indeed led to the establishment of the trained activity patterns in the brains of study participants. Regarding electrophysiological data, both the studies of Dohrmann et al. [30] and Crocetti et al. [29] did not include resting-state EEG measurements before and after the whole training period and did not obtain EEG data in the follow-up measurements. Instead, they focused their analysis on data obtained during the training phase (before and after each training) where they reported rather unspecific *increasing trends* of the alpha/delta ratio over the course of sessions. In contrast to these previous reports, we considered resting-state EEG data obtained before and after the entire neurofeedback intervention to be more informative for objective changes in electrophysiological activity patterns as a long-term function of the treatment and thus to be more indicative of neurofeedback learning. Baseline resting-state EEG recording was thus performed in an environment essentially different from the training setting and some time before the actual start of the training period.

The comparison with the data obtained after all 15 sessions were completed showed that the trained alpha/delta ratio over the four training electrodes was higher after the training than before, suggesting a successful establishment of the desired frequency pattern. In this context, while a significant increase was found for EEG-WT, data from the EEG-NT condition did not show statistically significant effects in the anticipated direction (see Figure 2). A possible explanation for this inconsistency might be that, in the EEG-NT measurement, no clear and unambiguous instructions were given besides those to open and close the eyes and reduce muscle movements. During the 8 minutes of measurement, patients were thus free to contemplate whatever came to their minds which might have led to highly heterogeneous emotional reactions and evoked brain processes across measurements. In the other (EEG-WT) condition, however, an explicit instruction was given to the patients, asking them to focus on their tinnitus percept in order to control for unwanted tinnitus-suppressing activity, which has been found to occur continuously in the brains of chronic tinnitus patients (e.g., [31]). The enhanced focus on the tinnitus tone might have led to reduced heterogeneity of resting-state situations, thereby making them more comparable across the three measurement time points. Furthermore, the EEG used for neurofeedback training was also registered while a patient's tinnitus was clearly salient, thus making the altered EEG rhythms more likely to be reflected in this resting-state measurement condition. Taken together, we believe the significantly and stably increased alpha/delta ratio across the entire training period provides a valuable indication for the successful establishment of the trained frequency patterns.

### 4.2. Placebo Control

Despite the strong evidence for objective changes in brain activity, the lack of a placebo control group can certainly be considered a possible limitation of this study. We did not include a control group due to restrictions of time, infrastructure, and funding, as well as ethical reasons and other arguments discussed comprehensively in our previously published review [28]. To name the most important ones, we considered the investment on the part of the tinnitus patients, who received no monetary compensation for study participation, to be clearly out of proportion to justify placebo neurofeedback. Furthermore, we did not want to induce any form of expectation as to whether a subject believed themselves to be in the sham or verum neurofeedback group. Strehl et al. [27] have suggested that absent success after the first training sessions may automatically evoke misguided ideas on the part of patients to be assigned to the placebo group. This could negatively affect motivation and further treatment success regardless of what group the patients have in fact been allocated to. In a comparison with previously performed studies, the publication of Crocetti et al. [29] also does not mention the inclusion of a control group. Furthermore, even though Dohrmann et al. [30] reported the use of an active control group that worked with auditory frequency discrimination training, the legitimation of this group in the comparison to the rather specific neurofeedback setting remains unclear. In addition, the article of Hartmann et al. [61] should be mentioned in this context. This group performed an alpha neurofeedback training with chronic tinnitus patients and compared their results to a TMS and a sham-TMS condition. Without the use of a specific placebo neurofeedback control group, they could show that alpha power increased exclusively for the neurofeedback group.

However, especially in the field of tinnitus treatment, patients often enter a trial with moderately hopeful expectations as they have already endured a variety of disappointing treatment attempts on their own. This circumstance greatly increases the risk for placebo effects of any intervention, and unspecific effects of the training thus have to be considered and discussed [62]. Therefore, our data analysis attached great importance to minimizing the risk for these unspecific effects of neurofeedback training. In particular, our data analysis closely followed the considerations of Gruzelier [63] about specificity of neurofeedback treatments. The author suggested that three distinct forms of specificity have to be fulfilled in order to label a neurofeedback intervention successful: frequency band specificity (effects in the trained frequency bands and only in these bands), topographical specificity (effects over the trained electrodes and only in these locations), and outcome specificity (correlations between changes in brain activity and analyzed behavioral outcomes) [63]. It will be discussed in the following section whether our data can support these three types of specificity.

### 4.3. Specificity of Effects

Regarding *frequency band specificity*, the data of this study indeed suggested specific effects in the trained frequency bands. As already discussed above, the alpha/delta ratio measured over the four training electrodes increased due to the intervention and remained on a stable high level in the follow-up period. Furthermore, we did not find any changes in other standard frequency bands which clearly speaks in favor of frequency band specificity for the applied neurofeedback protocol.

*Topographical specificity*, on the other hand, could not be confirmed with the data of this clinical study. The repeated-measure mixed model analysis of variance did suggest significant ratio effects over time not only for the four training electrodes but also over all 65 electrodes used for pre-, post-, and follow-up measurements. The neurofeedback protocol used in this study, therefore, did not only affect frequency band power in the vicinity to trained electrodes specifically but led to a global effect across the whole brain. This finding, however, is not unexpected since neurofeedback on the basis of activity measured with a limited number of electrodes on the scalp is generally considered to be unspecific, leading to widespread effects across the whole brain [64]. Unfortunately, neither Dohrmann et al. [30] nor Crocetti et al. [29] provided any information about possible activity changes on electrodes besides the trained ones. Furthermore, even Gruzelier [63] discusses the general possibility of topographically unspecific effects of surface-based neurofeedback. If the brain is seen as a holistic functional network rather than an aggregation of several strictly localized centers, topographically widespread effects of frequency band neurofeedback training should come as no surprise [63]. Also in the context of tinnitus, the view has recently shifted from the localized perspective to a more holistic concept with several proposed models aimed at describing the different (sub-)networks that contribute to the tinnitus percept (e.g., [33, 65]).

Finally, regarding *outcome specificity*, correlation analyses between difference scores of tinnitus and electrophysiological measures revealed an inconsistent picture. Meaningful negative correlations regarding the trained frequency bands could only be found with the changes in the Tinnitus Questionnaire. While a decrease of TQ scores was related to an increase of the alpha/delta ratio of EEG-WT on the trend level, the relation with increments in the rewarded individual alpha band was found to be statistically significant. It thus seems as if the increase in alpha was the driving force behind the improvements of tinnitus-related distress as measured with TQ. However, since also THI-measured distress as well as tinnitus loudness declined over the course of the training, we expected these changes to be related with electrophysiological measures as well, which was not the case.

Inconsistencies were also reported in the previous studies with comparable neurofeedback protocols as Dohrmann et al. [30] found electrophysiological measures to be correlated only with tinnitus loudness but not distress, while Crocetti et al. [29] reported findings to the exact opposite. In our study, Figures 3 and 4 provide a deeper look into the patterns of responder and nonresponder individuals in the study sample. In doing so, obvious neurofeedback responders can be identified as patients who were able to improve their alpha/delta ratio (increase their alpha, decrease their delta) and show reduced tinnitus symptoms (cases in the upper left quadrant for the ratio and IAF or in the lower left for delta). In contrast, obvious non-responders are also visible as cases unable to alter electrophysiological activity in the desired direction and not showing any changes or even increases in tinnitus symptoms (cases in the lower right quadrant for the ratio and IAF or the upper right for delta). There are, however, also examples of inconsistent cases. Several patients indicated having substantially benefited from the training and reported their tinnitus-related symptoms to be significantly lower, yet they did not show any EEG training effects (cases in the lower left quadrant for the ratio and IAF and in the upper left quadrant for delta). Others proved to be extremely successful in adjusting their brain activity in the intended direction over the course of training but did not report any or hardly any noticeable changes in tinnitus symptoms (cases in the upper right quadrant for the ratio and IAF and in the lower right for delta). Thus, even a superficial visual impression of our data already suggests a considerable amount of variability in the set. While the group in its entirety seems to have benefited from the neurofeedback application on average, a closer inspection of the results suggests a more complex picture in that we have identified a considerable amount of behavioral and/or electrophysiological non-responders. Therefore, a thorough future analysis of responder and non-responder groups would certainly prove fruitful in order to fathom the characteristics of certain subgroups and pave the way for better-suited neurofeedback protocols for each of them. These advanced analyses of data obtained in the scope of this study should also include considerations about the clinical relevance of observed difference scores (e.g., [66]) and will thus be discussed elsewhere.

## 5. Conclusion

To sum up, the neurofeedback protocol with individualized reward frequency bands discussed in this article can be considered a good option in the treatment of chronic tinnitus. We base this statement on the result that the distress of tinnitus sufferers was significantly and sustainably reduced and that a temporary effect for tinnitus loudness was also found. In order to influence the intensity of the percept in a sustainable way, a higher frequency (2-3 sessions a week) and longer training sessions (min 20 minutes) might be recommended. Even though unspecific effects are difficult to exclude due to the lack of a placebo control group, this study significantly extends current work in the field by carrying out data analysis with utmost care. Compared to most neurofeedback studies to date that did not take the unspecific effects of this intervention into account, we were able to demonstrate the frequency band specificity of our protocol. Even though the training did not lead to topographically specific but rather global effects, this result speaks in favor of specific effects of the intervention. Neurofeedback-induced changes in tinnitus-related symptoms seem to be mainly driven by an increase in alpha rather than a decrease in delta power, and the relationship with the trained bands was strongest for distress measured with the TQ (see Figure 4). In the light of the TCD model and the SLIM, this finding suggests that tinnitus distress as well as loudness are closely related to inhibitory activity in auditory areas reflected in the alpha band. If activity in inhibitory neurons is fostered with neurofeedback training and thus the disturbed excitatory/inhibitory balance readjusted, the tinnitus percept seems to be softened and its distressing component weakened. However, as has been shown, individual reactions to the neurofeedback training are heterogeneous and thus do not speak in favor of outcome specificity on the whole. More comprehensive analysis of responder and non-responder data will prove to be crucial in future studies in order to establish individually based neurofeedback. These insights would contribute in the pursuit of the long-term goal of developing training protocols catering to the specific needs of each tinnitus patient.

## Figures and Tables

**Table 1:** Demographics, health, and tinnitus characteristics of the study sample.

	Mean	SD	Median	Min	Max
Age	46.29	12.22	44	24	71
Mean hearing loss (dB)	7.54	8.25	4.4	0	22.8
Tinnitus duration (months)	78.92	74.63	40	18	312
Age of onset	39.75	14.66	39	14	67
Tinnitus loudness	53.25	19.57	50	20	95
Tinnitus distress (THI)	29.33	14.7	27	4	56
Tinnitus distress (TQ)	23.75	11.63	23	6	45
BDI sum score^a^	6.29	4.34	7	0	13
BAI sum score^a^	7.12	5.77	6.5	0	21
WHOQOL-BREF domain 1: physical health^b^	76.49	14.48	79	43	100
WHOQOL-BREF domain 2: psychological health^b^	69.97	15.78	69	42	96
WHOQOL-BREF domain 3: social relationship^b^	66.32	19.73	67	25	100
WHOQOL-BREF domain 4: environment^b^	81.51	11.28	84	62	100
WHOQOL-BREF global value^b^	67.19	18.36	62	25	100
SCL-K-9^c^	0.72	0.71	1	0	3
SF-36: mental health^e^	45.79	9.46	47	22	60
SF-36: physical health^e^	53.38	6.76	55	35	60

Note: SD: standard deviation. ^a^Sum scales (0-84) measuring severity of depressive/anxiety symptoms. ^b^Scaled sum scores (0-100) indicating quality of life in specific domains or globally. ^c^Mean over all items (0-4) measuring general psychological strain. ^e^Normed sum scales (*M* = 50, SD = 10) indicating mental/physical disability; higher values indicate less disability.

**Table 2:** Pearson correlation between tinnitus and health questionnaires.

	THI	TQ	Loudness
BDI sum score	0.75^∗∗∗^	0.79^∗∗∗^	0.48
BAI sum score	0.34	0.41	-0.03
SCL-K-9	0.47	0.56^∗^	0.30
WHOQOL-BREF domain 1: physical health	-0.65^∗∗^	-0.42	-0.37
WHOQOL-BREF domain 2: psychological health	-0.63^∗∗^	-0.55^∗^	-0.52^∗^
WHOQOL-BREF domain 3: social relationship	-0.30	-0.24	-0.19
WHOQOL-BREF domain 4: environment	-0.16	-0.11	-0.13
WHOQOL-BREF global value	-0.51^∗^	-0.25	-0.20
SF-36 physical health	-0.43	-0.22	0.02
SF-36 mental health	-0.69^∗∗^	-0.66^∗∗^	-0.45

Note: Pearson correlation coefficient corrected for multiple comparisons with the method of Benjamini and Hochberg [59]. ^∗^*p* < .05; ^∗∗^*p* < .01; ^∗∗∗^*p* < .001.

**Table 3:** Primary outcome variables of the study group.

	t1	t2	t3	t4
THI	29.33 (14.70)	23.92 (12.71)	24.83 (12.48)	24.75 (16.48)
TQ	23.75 (11.63)	21.62 (12.03)	21.54 (11.18)	20.58 (12.81)
Loudness	53.25 (19.57)	43.67 (22.42)	51.67 (22.00)	55.46 (17.28)
Ratio EEG-NT	0.955 (0.044)	0.959 (0.033)	0.965 (0.040)	
Ratio EEG-WT	0.961 (0.042)	0.978 (0.044)	0.968 (0.041)	

Note: values are mean (SD).

**Table 4:** Results of the repeated-measure mixed model ANOVA and a priori-defined contrasts for primary outcome variables.

	*χ* ^2^	*t*	df	*p*	Effect size *r*
THI					
ANOVA	9.18^∗^		3	0.027	
t1-t2		-2.76^∗^	69	0.011	0.32
t1-t3		-2.30^∗^	69	0.037	0.27
t1-t4		-2.34^∗^	69	0.033	0.27
TQ					
ANOVA	5.24		3	0.155	
t1-t2		-1.48	69	0.214	0.18
t1-t3		-1.54	69	0.192	0.18
t1-t4		-2.21^∗^	69	0.046	0.26
Loudness					
ANOVA	12.4^∗∗^		3	0.006	
t1-t2		-2.74^∗^	69	0.012	0.31
t1-t3		-0.45	69	0.978	0.05
t1-t4		0.63	69	0.794	0.08

Ratio EEG-NT					
ANOVA	3.54		2	0.170	
t1-t2		0.72	46	0.475	0.11
t1-t3		1.86^∗^	46	0.069	0.26
IAF EEG-NT					
ANOVA	0.99		2	0.610	
t1-t2		0.83	46	0.409	0.12
t1-t3		0.86	46	0.394	0.13
Delta EEG-NT					
ANOVA	1.24		2	0.539	
t1-t2		0.15	46	0.885	0.02
t1-t3		-0.87	46	0.390	0.13

Ratio EEG-WT					
ANOVA	7.77^∗^		2	0.021	
t1-t2		2.83^∗∗^	46	0.007	0.39
t1-t3		1.21	46	0.234	0.17
IAF EEG-WT					
ANOVA	0.51		2	0.776	
t1-t2		0.69	46	0.494	0.10
t1-t3		0.24	46	0.811	0.04
Delta EEG-WT					
ANOVA	5.74		2	0.057	
t1-t2		-2.42^∗^	46	0.020	0.34
t1-t3		-1.13	46	0.263	0.16

Note: *p* values of contrast analysis are Bonferroni corrected and one-tailed. ^∗∗^*p* < .01; ^∗^*p* < .05.

**Table 5:** Results of the repeated-measure mixed model ANOVA for control comparisons.

	*χ* ^2^	df	*p*
Standard bands			
EEG-NT			
Delta	6.60^∗^	2	0.037
Theta	1.20	2	0.549
L-Alpha	1.24	2	0.538
U-Alpha	1.05	2	0.591
Alpha	1.07	2	0.587
Beta1	0.17	2	0.917
Beta2	2.41	2	0.300
Beta3	1.82	2	0.402
Gamma	1.00	2	0.607
EEG-WT			
Delta	8.70^∗^	2	0.013
Theta	1.49	2	0.474
L-Alpha	0.25	2	0.881
U-Alpha	0.17	2	0.916
Alpha	0.70	2	0.706
Beta1	0.36	2	0.836
Beta2	0.15	2	0.925
Beta3	3.11	2	0.211
Gamma	4.25	2	0.119

Ratio over all electrodes			
EEG-NT	9.67^∗∗^	2	0.008
EEG-WT	9.60^∗∗^	2	0.008

Control for age group			
THI	4.57	4	0.335
TQ	8.24	4	0.083
Loudness	1.39	4	0.846
Ratio EEG-NT	7.66	3	0.054
Ratio EEG-WT	1.52	3	0.677

Note: ^∗∗^*p* < .01; ^∗^*p* < .05.

**Table 6:** Pearson correlation between changes in tinnitus measures and trained EEG frequency band.

	THI	TQ	Loudness
EEG-NT			
Ratio	0.12	-0.03	0.08
IAF	0.25	-0.12	-0.25
Delta	0.10	-0.10	-0.28

EEG-WT			
Ratio	-0.12	-0.34^+^	-0.14
IAF	-0.06	-0.40^∗^	-0.11
Delta	0.09	-0.10	0.06

Note: Pearson product-moment correlation coefficients of difference scores (t2-t1). ^∗^*p* < .05 (one-tailed); ^+^*p* < .1 (one-tailed).

## Data Availability

The data used to support the findings of this study are available from the corresponding author upon request.
